# Efficacy and safety of chemoradiotherapy plus immune checkpoint inhibitors for the treatment of locally advanced cervical cancer: a systematic review and meta-analysis

**DOI:** 10.3389/fimmu.2024.1459693

**Published:** 2024-09-16

**Authors:** Zhihong Zhao, Jian Ruan, Minjie Fang, Jingwen Liu, Guixiang Liao

**Affiliations:** Department of Radiation Oncology, Shenzhen People’s Hospital, The Second Clinical Medical College of Jinan University, Shenzhen, China

**Keywords:** chemotherapy, randomized controlled trials, cervical cancer, radiotherapy, immune checkpoint inhibitors

## Abstract

**Background:**

Radiotherapy plus concurrent chemotherapy is a standard method for treating locally advanced cervical cancer (LACC). Immune checkpoint inhibitors (ICIs) are widely applied in the treatment of recurrent cervical cancer, metastatic cervical cancer or LACC. The efficacy and safety of radiotherapy plus immunotherapy for LACC require further investigation. The objective of this review and meta-analysis was to analyze the efficacy and safety of concurrent chemoradiotherapy (CCRT) combined with ICIs for treating LACC on the basis of the results of randomized controlled trials (RCTs).

**Methods:**

We comprehensively searched electronic databases to identify RCTs that focused on CCRT plus ICIs for LACC treatment. The outcomes included the objective response rate (ORR) and progression-free survival (PFS), overall survival (OS) and adverse events (AEs). A standard method for systematic review and meta-analysis was used. Review Manager 5.4 was used for data combination and analyses.

**Results:**

Three RCTs involving 1882 participants with LACC were identified and included in the systematic review and meta-analysis. CCRT plus ICIs improved the rates of PFS (hazard ratio [HR]: 0.76, 95% confidence interval [CI]: CI: 0.64, 0.91, *P* = 0.002) and OS (HR: 0.7695% CI (95% CI 0.58–0.99, *P* = 0.04) in patients with LACC. Compared with the control group, the CCRT plus immunotherapy group had an increased ORR (OR: 1.37, 95% CI: 1.02,1.85, *P*=0.04). The two methods had similar rates (HR=1.99, 95% CI: 0.99, 1.43; *P*=0.07) of treatment-related grade 3 or higher AEs. The CCRT plus immunotherapy group had a higher rate than did the control group (HR: 2.68, 95% CI: 1.38, 5.21; *P=*0.004) in terms of any grade immunotherapy-related AEs.

**Conclusions:**

CCRT plus ICIs is efficacious and safe for the management of LACC. The addition of ICIs to CCRT improved the rates of PFS and OS in patients with LACC. The adverse effects of immunotherapy-related AEs should be strictly examined and managed in a timely manner.

## Introduction

Cervical cancer is the fourth most common malignant tumor in the world and poses a serious threat to human health. Cervical cancer is the fourth leading cause of cancer death among women. According to statistics, there are approximately 600000 new cases of cervical cancer worldwide each year, with 90% of cases occurring in low- and middle-income countries (1–3). Early cervical cancer can be cured through surgery, but approximately half of patients are locally advanced at initial diagnosis (4–6). Concurrent chemoradiotherapy (CCRT) based on cisplatin combined with brachytherapy is the standard treatment for locally advanced cervical cancer (LACC). However, after completion of CCRT, the prognosis of these patients remains poor, with a 5-year OS rate of approximately 65–70% and nearly 40% of patients experience recurrence or metastasis (7–9). Reducing distant metastasis and improving the long-term survival rate of patients with LACC remain urgent clinical issues that need to be addressed. Immune checkpoint inhibitors (ICIs) (such as cytotoxic T-lymphocyte-associated protein 4 (CTLA-4), programmed cell death-1 (PD-1), and PD-L1 inhibitors) have emerged as important strategies for various cancers (10, 11). Mounting evidence indicates that immunotherapy has good effectiveness and safety in treating malignant tumors such as melanoma (12), lung cancer (13), and liver cancer (14). In recurrent, metastatic cervical cancer (R/M CC), the Keynote-826 trial demonstrated that immunotherapy is safe and effective in the treatment of R/M CC, improving OS and PFS (15, 16). Some studies have applied ICIs in LACC treatment and confirmed that immunotherapy plays a certain antitumor role, with compelling results (17–19).

However, there is still a lack of sufficient clinical evidence on the efficacy and safety of CCRT combined with ICIs in LACC patients. In this systematic study and meta-analysis, we systematically elucidated the efficacy of CCRT combined with immunotherapy in LACC patients on the basis of published randomized controlled trials (RCTs).

### Objectives and research question

Therefore, this review aimed to summarize the clinical trials that have focused on CCRT combined with ICIs for the management of LACC.

## Methods and materials

### Study registration

This meta-analysis protocol was registered on PROSPERO (ID: 560803). This study was conducted in accordance with the Preferred Reporting Items for Systematic Reviews and Meta-Analyses (PRISMA) statement.

### Search strategy

We conducted a systematic search of the Web of Science, PubMed, EMBASE, ClinicalTrial, ScienceDirect and Cochrane Library databases. The search terms included cervical cancer, immunotherapy, checkpoint inhibitor, radiotherapy and chemotherapy. The latest search was conducted on 22 June 2024. First, a repeated evaluation of the literature obtained from the search was conducted. After removing duplicates, a reviewer screened the titles of the studies to identify potentially suitable studies. Two reviewers subsequently independently screened the records on the basis of the abstracts/full texts. If there was any disagreement regarding the included literature, it was resolved through discussion.

### Participants, interventions, and comparator

Patients aged >18 years who had an LACC diagnosis confirmed by pathology were included. Patients who experienced recurrence were excluded.

#### Intervention

##### Treatment group

Patients who received CCRT with concurrent immunotherapy.

##### Control group

Patients who received CCRT without concurrent immunotherapy.

#### Outcomes

##### Primary outcomes

Objective response rate (ORR).

Progression-free survival (PFS).

Overall survival (OS).

##### Secondary outcomes

Adverse events (AEs) included all-grade treatment-related AEs, treatment-related grade 3 or higher AEs, all-grade immunotherapy-related AEs (irAEs), grade 3 or higher irAEs and individual toxicity ≥grade 3.

Furthermore, the inclusion criteria were as follows: (1) studies including women diagnosed with cervical cancer by pathology; (2) studies including at least 20 patients; (3) studies published in English since 2015; (4) studies reporting safety or survival data; and (6) RCTs. The exclusion criteria were as follows: comments, editorials, guidelines, opinions, letters, and meeting summaries.

### Quality assessment

The Cochrane tool was applied to assess the quality of the RCTs (20). The bias assessment included selection bias, performance bias, detection bias, attrition bias, reporting bias and other bias assessments; these items were evaluated by two independent reviewers, and any disagreements were resolved by discussion among the review group.

### Data extraction

All the data were extracted via standardized methods. The extracted information included the first author of the study, publication year, sample size, treatment method and medication, Eastern Cooperative Oncology Group (ECOG) performance status score, Federation International of Gynecology and Obstetrics (FIGO) stage, histology, nodal involvement, follow-up times and results of interest (ORR, PFS, OS, and AEs). The secondary outcomes of interest included locoregional progression events, distant progression events and toxicity. Data on the outcomes of interest were extracted by two independent reviewers. All reviewers resolved any disagreements through discussion.

### Statistical analysis

Statistical analysis was conducted via RevMan 5.4 (Nordic Cochrane Centre). The risk ratio (HR) and its 95% confidence interval (CI) were used to describe survival outcomes. The odds ratio (OR) and its 95% CI were used to evaluate AEs and ORRs. I^2^ was used to evaluate heterogeneity, and 25%, 50%, and 75% values were considered low, medium, and high, respectively (21). If I^2^ was <25%, a fixed-effects model was used for data analysis; otherwise, a random-effects model was used. A P value<0.05 was considered statistically significant. Subgroup analysis and sensitivity analysis were subsequently conducted. Egger and Begg tests were used to evaluate publication bias (22).

## Results

### Study selection and characteristics

**Figure 1:**
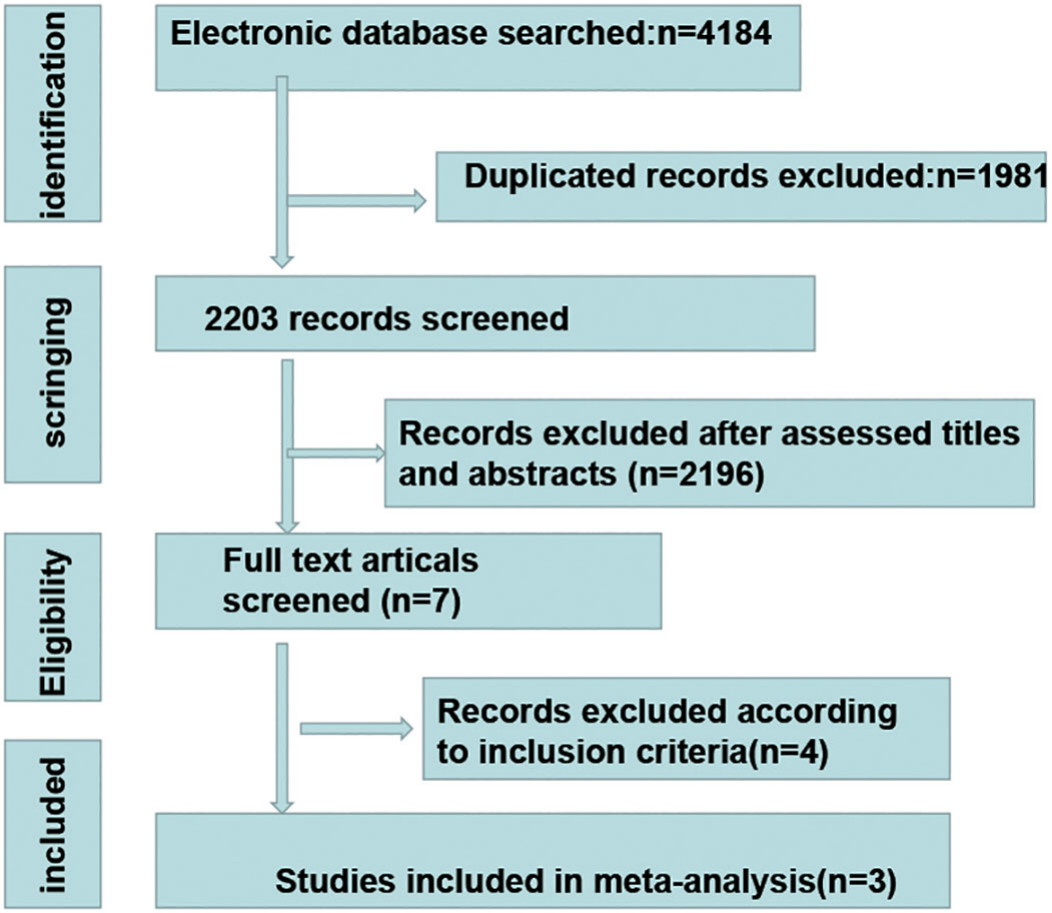
The process of study selection.

**Figure 2:**
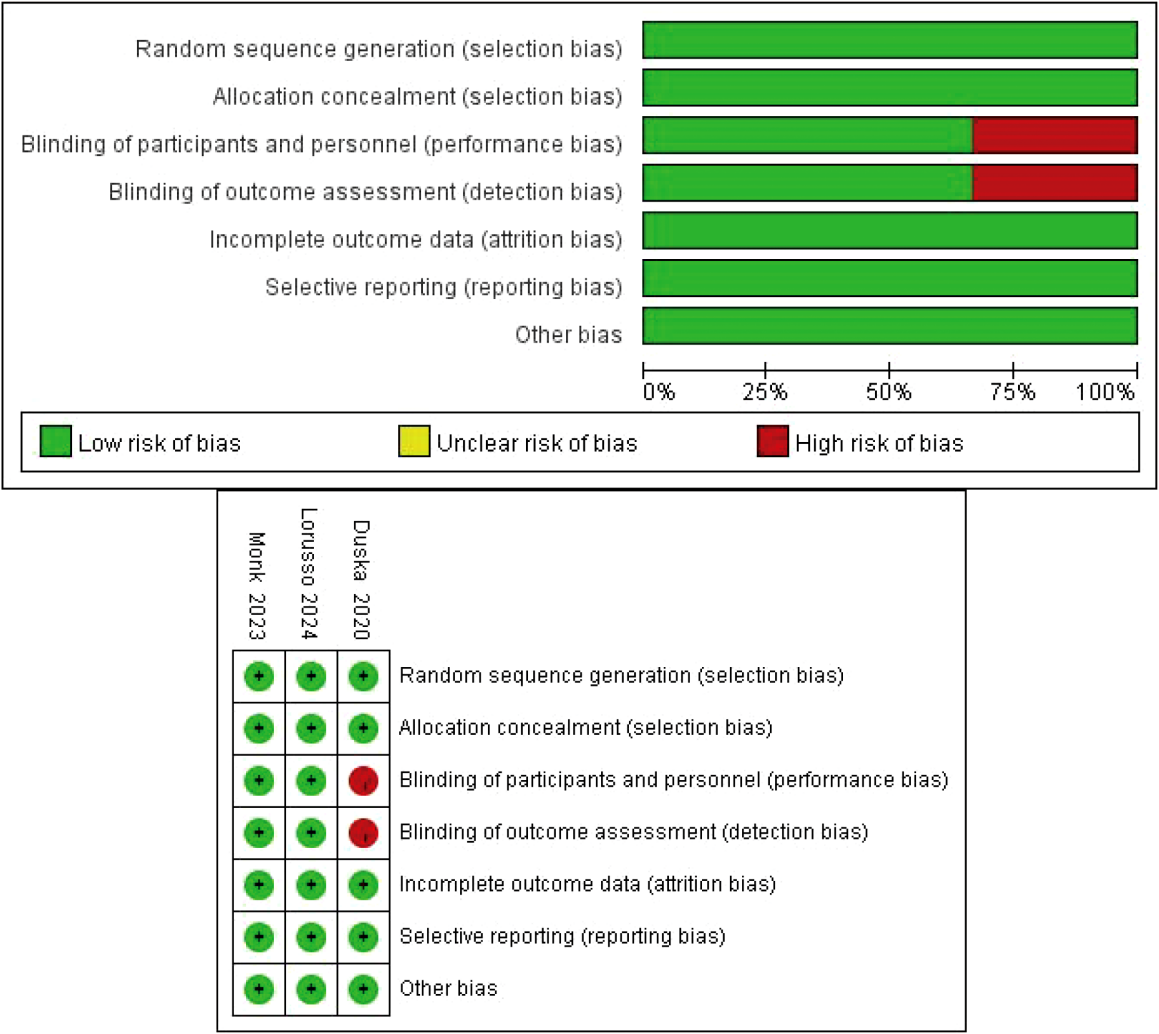
Risk of bias assessment.

Overall, three RCTs, involving 1882 participants with LACC, were included in this review and meta-analysis (23–25). A total of 942 patients were included in the CCRT plus ICIs group, and 940 patients were included in the control group. The follow-up time ranged from 4.6 months to 18.5 months, and 1336 patients had an ECOG performance status score of 0. A total of 544 patients had an ECOG performance status score of 1, and two patients had an ECOG performance status score of 2. A total of 1569 patients had cervical squamous cell carcinoma, 768 patients had FIGO stage IB2-IIB disease, and 1480 patients had positive lymph nodes. The basic information of the included studies is shown in **Table 1**. The selection process is outlined in **Figure 1**, and the risk of bias evaluation is presented in **Figure 2**.

**Table 1:** the basic information of included randomized controlled trials(RCTs).

Study	Design	Treatment	Case	Median age	ECOG Score 0/1/2	FIGO stage I-II/III-IV	Histology Non-squamous^#^/squamous	Nodal status N0/N+	Followed-up time(m)
Lorusso et al.	Phase 3,double-blind	CCRT plus pembrolizumab	529	49 (40–57)	380/149/0	235/294	96/433	84/445	17.9
		CCRT	531	50 (41–59)	397/134/0	227/304	80/451	93/438	17.9
Duska et al.	phase2,open label	CCRT plus pembrolizumab	28	49 (28-74)	21/7/0	20/8	4/24	12/16	4.6
		CCRT following pembrolizumab	24	49 (28-74)	18/5/1	21/3	5/19	13/11	9.2
Monk et al.	phase3,double-blind	CCRT Plus Durvalumab	385	50 (41-57)	265/119/1	135/250	63/322	106/279	18.5
		CCRT	385	48 (40–57)	255/130/0	130/255	65/320	94/291	18.4

CCRT, concurrent chemoradiotherapy; ECOG, Eastern Cooperative Oncology Group; FIGO, Federation International of Gynecology and Obstetrics;

^#^ Includes adenocarcinoma and adenosquamous carcinoma.

### Objective response rate

**Figure 3:**
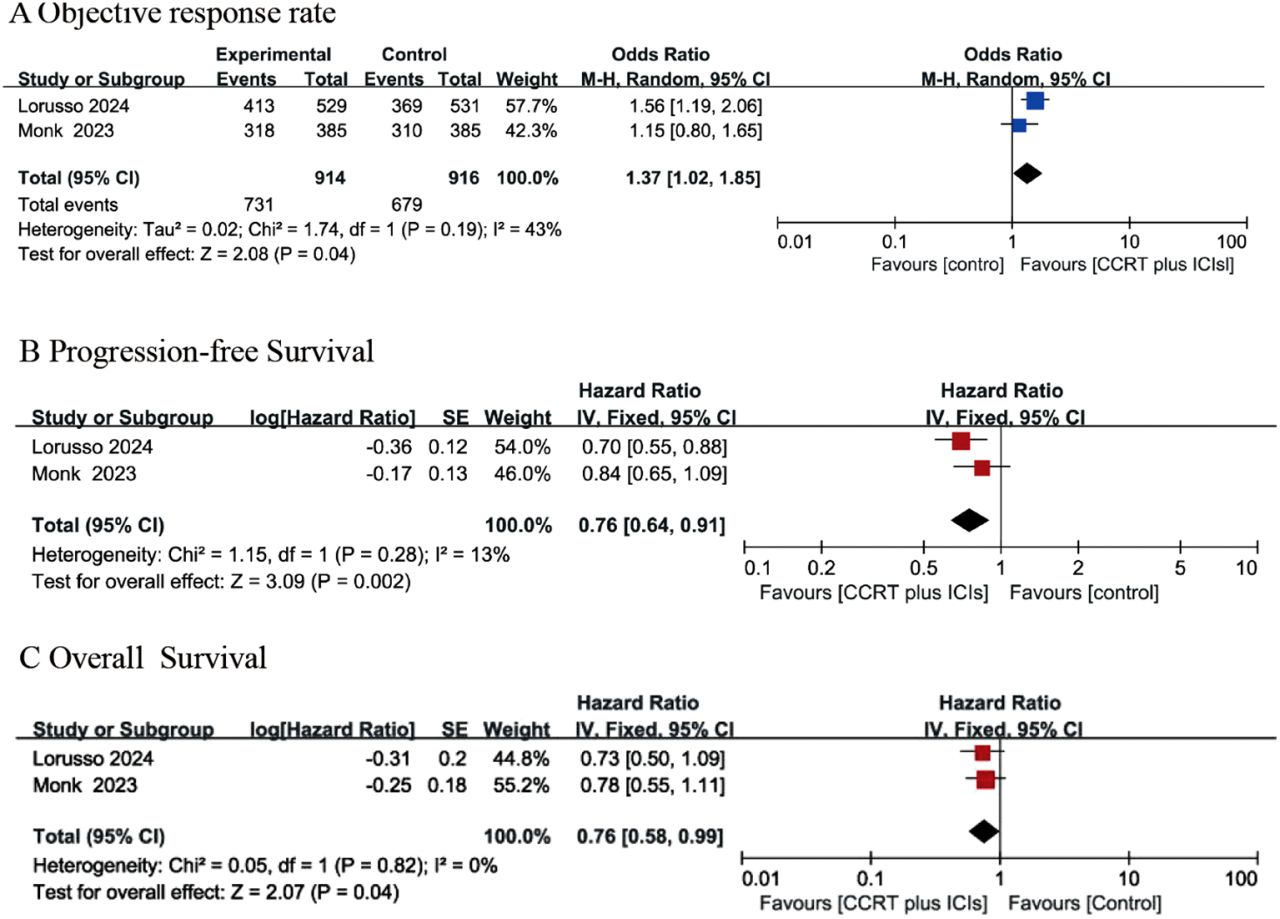
Forest plots for objective response rate **(A)**, progression-free survival **(B)** and overall survival **(C)** between concurrent chemoradiotherapy (CCRT) plus ICIs and control group.

Two RCTs (24, 25) described the ORR. As described by Lorusso et al. (24), the ORR was 79% and 76% in the intervention group and the control group, respectively. Monk et al. (25) indicated that the ORR was 83% and 81% in the CRT plus immunotherapy group and the control group, respectively. Pooled data from the two studies (24, 25) indicated that the CCRT with concurrent immunotherapy group had an increased ORR compared with that of the control group (OR: 1.37, 95% CI: 1.02, 1.85), and the *P* value was 0.04 (**Figure 3A**). A random-effects model was used for analysis because of high heterogeneity (Chi^2^ = 1.74, I^2^ = 43%, P=0.19).

### Progression-free survival

Only two RCTs (24, 25) reported PFS. Lorusso et al. (24) indicated that the PFS rates were 22% and 29% in the intervention group and the control group, respectively, with an HR of 0·70 (95% CI: 0·55–0·89). Monk et al. (25) reported that the 12-month PFS rate was 76·0% in the intervention group and 73·3% in the control group, with an HR of 0·84 (95% CI 0·65–1·08). In summary, the results of two RCTs (24, 25) suggested that the CCRT with concurrent immunotherapy group had an improved PFS rate compared with that of the control group (HR: 0.76 (95% CI: 0.64, 0.91), *P* value=0.002), as shown in **Figure 3B**, and the analysis revealed no significant heterogeneity (Chi^2^ = 1.15, I^2^ = 13%, P=0.28).

### Overall survival

Two RCTs reported results on OS (24, 25). Lorusso et al. (24) indicated that a total of 44 (8%) patients in the intervention group and 59 (11%) patients in the control group experienced OS events, with an HR of 0·73 (95% CI: 0·49–1·07). Monk et al. (25) reported that the death rate was 15% in the intervention group and 19% in the control group, with an HR of 0·78 and a 95% CI of 0.55-1.10. The combined data (24, 25) indicated that the CCRT with concurrent immunotherapy group had a favorable OS rate compared with that of the control group, with an HR of 0.76 (95% CI 0.58-0.99) and a *P* value of 0.04. A fixed-effects model was used for analysis because there was no heterogeneity (Chi^2^ = 0.05, I^2^ = 0, P=0.82), as presented in **Figure 3C**.

Local progression events and distant progression events

Only Monk et al. described local progression events. There were 42 and 40 local progression events in the CCRT with concurrent immunotherapy group and the control group, respectively. The analysis revealed that the OR for local progression events was 1.06 (95% CI: 0.67, 1.67), and the P value was 0.82. Only Monk et al. provided data on distant progression events. There were 52 and 69 distant progression events in the experimental group and the control group, respectively. The pooled data indicated that the OR for distant progression events was 0.71 (95% CI: 0.48, 1.05), and the P value was 0.09.

### Adverse events

Three RCTs (23–25) described any grade of treatment-related AE. The data revealed that the two strategies had comparable grades of treatment-related AE (HR: 1.22, 95% CI: 0.55, 2.67; *P*=0.62) (**Supplementary Figure S1**). For treatment-related Grade 3 or higher AEs, the pooled data indicated that the two methods had similar rates (HR=1.99, 95% CI: 0.99, 1.43; *P*=0.07), but CCRT plus ICIs tended to have a higher rate (**Supplementary Figure S2**). With respect to any-grade irAEs, the CCRT combined with immunotherapy group had a higher rate compared to that of control group (HR: 2.68, 95% CI: 1.38, 5.21, *P*=0.004), and a random-effects model was used for analysis (I^2^ = 80, *P*=0.007) (**Supplementary Figure S3**). In terms of grade 3 or higher immunotherapy-related treatment AEs, Duska et al. (23) reported one case of grade 3 hyperthyroidism in the control group and no AEs in the CRT with concurrent immunotherapy group. As described by Lorusso et al. (24), the incidence of grade 3 or higher immunotherapy-related AEs was 4% and 1% in the intervention group and the control group, respectively.

Two studies provided details of toxicities (24, 25). With respect to grade ≥3 nausea, anemia, diarrhea, a decreased white blood cell count, a decreased neutrophil count, neutropenia, leukopenia, a decreased platelet count, hyperthyroidism and colitis, comparisons between the CCRT with concurrent immunotherapy group and the control group are provided in **Table 2**. The two groups had similar rates of toxicity.

**Table 2:** Meta-analysis of Grade ≥3 toxicity between concurrent chemoradiotherapy plus immunotherapy and control group.

Items Grade ≥3 toxicity	No.Of Trials	Effect model	OR and Its 95% CI	Z value	P value	Heterogeneity		
						Chi^2^	I^2^ (%)	P
Nausea	2	Random-effect	1.16 (0.39,3.43)	0.26	0.79	1.43	30	0.23
Anaemia	2	Fixed-effect	1.28 (0.99,1.65)	1.89	0.06	0.19	0	0.66
Diarrhoea	2	Random-effect	2.46 (0.19,31.46)	0.69	0.49	3.26	69	0.07
Decreased white blood cell count	2	Fixed-effect	0.86 (0.67,1.10)	1.19	0.24	0.34	0	0.56
Decreased neutrophil count	2	Fixed-effect	0.92 (0.69,1.22)	0.56	0.57	0.53	0	0.47
Neutropenia	2	Random-effect	1.31 (0.85,2.03)	1.21	0.23	1.45	31	0.23
Leukopenia	2	Fixed-effect	1.13 (0.82,1.55)	0.74	0.46	0.43	0	0.51
Decreased platelet count	2	Random-effect	1.33 (0.55,3.23)	0.63	0.53	2.37	58	0.12
Hyperthyroidism	2	Fixed-effect	5.03 (0.59,43.10)	1.47	0.14	0.00	0	1.00
Colitis	2	Random-effect	2.10 (0.25,17.27)	0.69	0.49	1.90	47	0.17

### Sensitivity analysis and subgroup analysis

We conducted sensitivity and subgroup analyses on PFS on the basis of age (≥65 versus <65 years), type of radiotherapy plan design (intensity modulated radiation therapy (IMRT)/volumetric modulated arc therapy (VMAT) versus non-IMRT/VMAT), and FIGO stage (IB2-IIB versus III-IV). The results are provided in **Table 3**. In the subgroup of patients aged less than 65 years and radiotherapy plan design by the IMRT/VMAT, radiotherapy combined with immunotherapy improved PFS compared with that of the control group. In the subgroup of patients with other factors (such as an age >65 years, a non-IMRT/VMAT plan, and CCRT plus immunotherapy), the PFS rate was similar to that of the control group.

**Table 3:** Subgroup and sensitive analysis on progression-free survival.

Items	No. Of studies	Effects model	HR and Its 95% CI	Z value	P value	Heterogeneity		
						Chi^2^	I^2^ (%)	P
Age<65	2	Fixed-effect	0.77 (0.64,0.92)	2.83	0.005	0.58	0	0.45
age≥65	2	Fixed-effect	0.72 (0.41,1.25)	1.17	0.24	0.92	0	0.34
IMRT/VMAT	2	Random-effect	0.76 (0.59,0.97)	2.22	0.03	1.59	37	0.21
Non-IMRT/VMAT	2	Fixed-effect	0.83 (0.53,1.31)	0.78	0.43	0.13	0	0.72
FIGO stage IB2-IIB	2	Fixed-effec	0.90 (0.67,1.19)	0.75	0.45	0.03	0	0.87
FIGO stage III-IV	2	Random-effect	0.78 (0.45,1.36)	0.87	0.38	9.80	90	0.002

FIGO, Federation International of Gynecology and Obstetrics; IMRT, Intensity Modulated Radiation Therapy; VMAT, Volumetric Modulated Arc Therapy.

### Publication bias

**Figure 4:**
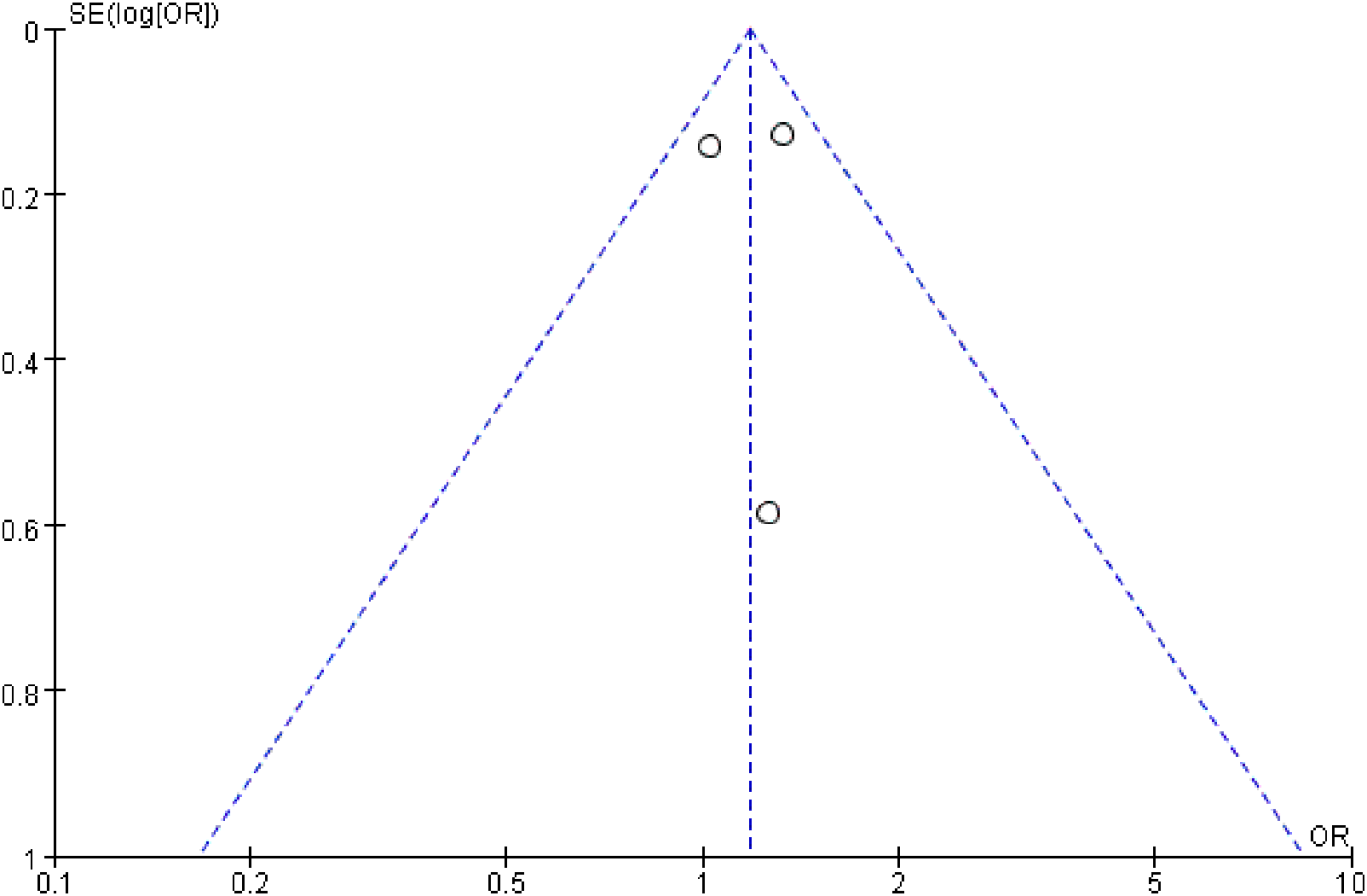
Publication bias was assessed by funnel plot of treatment-related grade 3 or higher adverse events.

A funnel plot of treatment-related grade 3 or higher AEs was used to evaluate publication bias in the included studies, and all the results within the 95% CIs revealed no significant publication bias (**Figure 4**). Because only three RCTs were included in this meta-analysis, we did not apply Egger or Begg tests for precise testing of publication bias.

## Discussion

### Summary of the main findings

Compared with the control group, patients who received CCRT with concurrent immunotherapy had longer OS (0.7695% CI (95% CI 0.58–0.99, *P=*0.04) and PFS (HR: 0.76, 95% CI: 0.64, 0.91, *P =* 0.002). The CCRT with concurrent immunotherapy group had an increased ORR compared with that of the control group (OR: 1.37, 95% CI: 1.02, 1.85, *P*=0.04). The two groups had similar rates of treatment-related Grade 3 or higher AEs (HR=1.99, 95% CI: 0.99, 1.43; *P*=0.07). CCRT plus ICIs was associated with a higher rate of any-grade irAEs (HR: 2.68, 95% CI: 1.38, 5.21; *P*=0.004).

Radiation therapy is often used to treat patients with cervical cancer. CCRT is the standard treatment for locally advanced nonsurgical cervical cancer (26). ICIs (CTLA-4, PD-1, or PD-L1) (27) are widely used to treat solid tumors (28), with the aim of utilizing host immunity to combat cancer, making them promising strategies for treating solid tumors. ICI treatment is an effective treatment method for cervical cancer (29). As confirmed in the KEYNOTE-826 phase III trial (16), the combination of pembrolizumab (an anti-PD-1 inhibitor) and first-line chemotherapy significantly improved the PFS of patients with R/M CC from 8.2 months to 10.4 months, and the 2-year OS rate also increased from 40.4% to 50.4%. Similarly, the midterm analysis of GOG-3016 revealed that (30), compared with chemotherapy, the PD-1 inhibitor cimipril monoclonal antibody improved OS in patients with R/M CC receiving second-line treatment. An increasing number of clinical trials have shown that ICIs have certain safety and efficacy in the treatment of cervical cancer.

In terms of the ORR, the ORR ranged from 76% to 83% in LACC patients in the included studies (24, 25). In patients with R/M CC who received pembrolizumab, the median ORR was 22.39%, ranging from 12.2% to 42% (31–33). In patients with cervical cancer receiving nivolumab, the ORR ranged from 15.8% to 93.8% (34). An excellent ORR (93.8%) was reported in the NICOL trial, in which patients with LACC were administered nivolumab in combination with CCRT (35). One trial (35) reported that the 2-year PFS rate was 75%. Our analysis revealed that CCRT with concurrent immunotherapy significantly increased the ORR (OR: 1.37, 95% CI: 1.02, 1.85; *P*=0.04). The combination of radiation therapy and immunotherapy for the treatment of cervical cancer is receiving widespread attention. Another study of stereotactic radiotherapy (SBRT) combined with atezolizumab (an anti-PD-L1 drug) in the treatment of R/M CC confirmed a median PFS of 4.5 months and a 6-month PFS rate of 46% [38]. The combination of CCRT with ICIs can significantly upregulate immune activation markers, leading to a significant increase in central and effector memory T cells and evidence of immune-modulating activity (36). The PRIMMO phase II trial investigated the efficacy of pembrolizumab combined with SBRT and immunomodulatory drug combinations in patients with R/M CC. The main ORR is 11.1%. The progression-free survival period is 4.1 weeks, and these drugs exhibit persistent and effective antitumor activity (37). A recent review revealed that ICIs improved PFS in patients with cervical cancer (HR, 0.68; 95% CI, 0.59–0.79) compared with the control treatment (38). These results are consistent with our finding that CCRT plus immunotherapy was associated with longer PFS than was the control treatment. A phase 2 RCT (39) reported that the PFS was 2.8 months and 1.9 months in patients with R/M CC who received ragolumab plus atezolizumab or atezolizumab, respectively. In another phase 2 trial of 27 patients with R/M CC treated with sintilimab in combination with chemotherapy, the ORR was 44.4%, and the median PFS was 5.2 months (40). Our subgroup analysis revealed that patients aged less than 65 years who received CCRT plus immunotherapy had longer PFS than did the controls. Moreover, in patients with the IMRT/VMAT radiotherapy plan, those who received CCRT plus immunotherapy also had a longer PFS. The development of new technologies in radiation therapy allows the delivery of higher doses, lowering toxicity and resulting in survival benefits (41). Some studies reported that VMAT combined with guided adaptive brachytherapy resulted in satisfactory PFS and OS in LACC patients (42). Moreover, compared with three-dimensional conformal radiotherapy (3D-CRT), the IMRT technique has a lower degree of radiotherapy toxicity in LACC patients (43). However, in a randomized trial, the effects of the two techniques (IMRT versus 3D-CRT) on relapse-free survival and disease-free survival did not differ, and whether IMRT treatment improved PFS compared with 3D-CRT needs further investigation (44).

In terms of OS, Lorusso et al. reported that the estimated 2-year OS rate was 87% in the intervention group and 81% in the control group and that the median OS was not reached in either group. In patients with cervical cancer who received nivolumab, the median OS ranged from 14.5 months to 21.9 months (34). In patients with R/M CC treated with pembrolizumab, the median OS ranged from 9.4 months to 11.2 months (31). Our study indicated that CCRT plus ICIs improved OS compared with the control treatment, which was consistent with the findings of previous studies (38).

Immunotherapy provides clinical benefits for cancer patients, and owing to its mechanism of action, it inevitably produces a series of side effects. These side effects may affect various organs or systems throughout the body, including the gastrointestinal tract, heart, skin, liver, endocrine system, and lungs (26). The occurrence and onset of immune-mediated adverse reactions depend on various factors, including cancer type, dosage, and ICI category, as well as patient-specific factors. For treatment-related grade 3 or higher AEs, the pooled data indicated that the two methods had similar rates (HR=1.99, 95% CI:0.99, 1.43, P=0.07), but CCRT plus ICIs had a higher rate, indicating that CCRT plus immunotherapy might increase toxicity. However, with respect to individual toxicity, such as ≥ grade 3 nausea, anemia, diarrhea, and a decreased white blood cell count, the two groups presented similar rates. A recent study indicated that ICIs combined with chemotherapy increased the incidence of all-grade AEs (HR 1.11 [1.09; 1.12]) but did not increase the treatment-related mortality rate (45). Toxicity can be safely managed with suitable methods (46). In most cases, ICI treatment can be closely monitored in the presence of mild irAEs. If level 3 toxicity occurs, the use of ICIs should be suspended. In the presence of level 4 toxicity, permanent cessation of ICI therapy is usually recommended; however, if endocrine function is abnormal due to immunity, it can be controlled through hormone replacement. The phase 2 studies included in this study indicate that pembrolizumab combined with CCRT is safe and effective in the treatment of LACC. Among the 52 patients included, 88% experienced treatment-related grade 2 or higher AEs, with approximately 22% experiencing at least one grade 4 AE and 23 experiencing at least one grade 3 AE. With the combination of atezolizumab and SBRT for patients with R/M CC, all patients completed the scheduled treatment with controllable tolerability. Among the most common grade 2 or above AEs, the most common were leukopenia (31%), fatigue (23%) and hypothyroidism (15%) (47). The PRIMMO phase II trial confirmed that pembrolizumab combined with SBRT treatment is safe and effective (30), which is consistent with our meta-analysis results.

### Limitations

This study has several limitations. First, only three RCTs were included in this meta-analysis, and one study did not report survival outcomes. This limited the statistical power. Second, the ICIs included in this meta-analysis were different drugs (pembrolizumab and durvalumab), which might explain the differences. Third, PD-L1 expression is an important biomarker for the prediction of treatment effects. Due to limited data, we did not conduct subgroup analysis on the basis of the PD-L1 level. In addition, this analysis included only fully published papers published in English, and studies with negative results might be ignored. Furthermore, in some analyses, high heterogeneity may exist, and some subgroup analyses do not yield positive results; these results should be interpreted with caution.

## Conclusions

Compared with the control treatment, CCRT plus ICIs significantly improved survival outcomes and increased the ORR. Similar rates of treatment-related grade 3 or higher AEs and toxicities were observed between the two groups. Moreover, large, well-designed RCTs are needed to further confirm the efficacy and safety of CCRT plus ICIs in LACC patients.

## Data Availability

The original contributions presented in the study are included in the article/**Supplementary Material**. Further inquiries can be directed to the corresponding authors.

## Supplementary material

Supplementary Figure 1 Forest plots for any grade of treatment adverse events between concurrent chemoradiotherapy (CCRT) plus ICIs and control group.

Supplementary Figure 2 Forest plots for treatment-related Grade 3 or higher adverse events between concurrent chemoradiotherapy (CCRT) plus ICIs and control group.

Supplementary Figure 3 Forest plots for all grade immunotherapy-related adverse events between concurrent chemoradiotherapy (CCRT) plus ICIs and control group. PRISMA Checklist.
