# Corrigendum: Efficacy and safety of chemoradiotherapy plus immune checkpoint inhibitors for the treatment of locally advanced cervical cancer: a systematic review and meta-analysis

**DOI:** 10.3389/fimmu.2024.1524791

**Published:** 2024-11-20

**Authors:** Zhihong Zhao, Jian Ruan, Minjie Fang, Jingwen Liu, Guixiang Liao

**Affiliations:** Department of Radiation Oncology, Shenzhen People’s Hospital, The Second Clinical Medical College of Jinan University, Shenzhen, China

**Keywords:** chemotherapy, randomized controlled trials, cervical cancer, radiotherapy, immune checkpoint inhibitors

In the published article, there was a mistake in the affiliations of all authors. The affiliations of all authors were displayed as “Department of Radiation Oncology, Second Clinical Medicine Centre, Jinan University, Shenzhen, China.”

The correct affiliations of all authors is “Department of Radiation Oncology, Shenzhen People’s Hospital, The Second Clinical Medical College of Jinan University, Shenzhen, China.’’

The authors apologize for this error and state that this does not change the scientific conclusions of the article in any way. The original article has been updated.

