# Supplementary figures and images for: Efficacy and safety of chemoradiotherapy plus immune checkpoint inhibitors for the treatment of locally advanced cervical cancer: a systematic review and meta-analysis
Source: Front Immunol. 2024 Sep 16;15:1459693. doi: 10.3389/fimmu.2024.1459693 (PMC11439685; doi:10.3389/fimmu.2024.1459693)

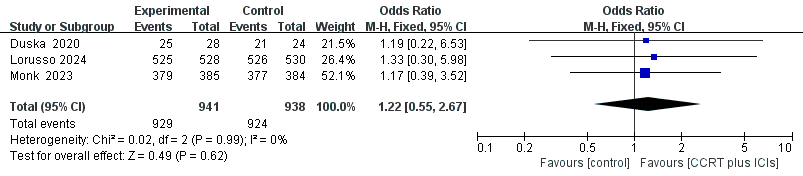

Supplement: Supplementary Figure 1 — Forest plots for any grade of treatment adverse events between concurrent chemoradiotherapy (CCRT) plus ICIs and control group. [file Image1.png]

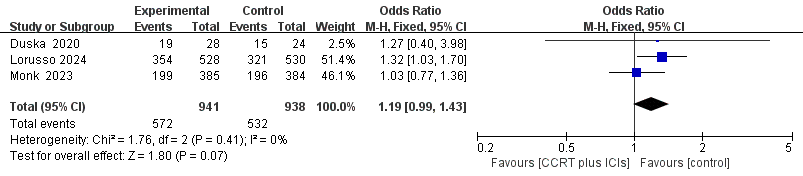

Supplement: Supplementary Figure 2 — Forest plots for treatment-related Grade 3 or higher adverse events between concurrent chemoradiotherapy (CCRT) plus ICIs and control group. [file Image2.png]

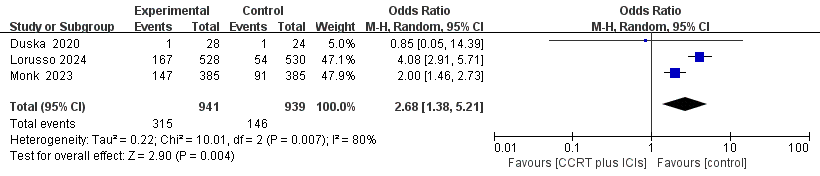

Supplement: Supplementary Figure 3 — Forest plots for all grade immunotherapy-related adverse events between concurrent chemoradiotherapy (CCRT) plus ICIs and control group. PRISMA Checklist. [file Image3.png]
